# Current News

**Published:** 1890-05

**Authors:** 


### Current News.

---

At the March meeting of the Chicago Dental Club it was voted to engage Dr. W. X. Sudduth to deliver a course of lantern lectures upon Biology during the first and second weeks of June.

---

On March 25 the Chicago College of Dental Surgery graduated a class of sixty. The whole class numbered seventy-one, but eleven of these failed to pass satisfactory examinations and consequently did not receive the much coveted diplomas. This is an indication of more thorough work on the part of the college and a determination not to graduate incompetent men.

Dental Education was the theme for discussion at the last meeting of the Chicago Dental Club. The paper of the evening was prepared by Dr. C. Stoddard Smith, of the Illinois State Board of Dental Examiners. The discussion was opened by Professor E. L. Clifford and Dr. E. S. Talbot.

---

The Faculty of the Chicago College of Dental Surgery are determined to have the "second" International Dental Congress held in Chicago in 1892 or 1893, and secure its control; at least it would seem so from the speeches made at their late commencement banquet.

---

Dr. Crouse has been invited by the Chicago Dental Club to present the interests of the "Dental Protective Association" at the April meeting. He is doing an unselfish work and should be encouraged to go on. The very least that the members of the profession can do is to join the Association. Mail him that *ten dollars* at once and save yourself from the clutches of a worse enemy than Josiah Bacon.

---

The commencement exercises of the American College of Dental Surgery, Chicago, were held on March 25, and a class of twenty-nine were launched upon the troubled sea of professional life.

---

The University Dental College, Chicago, held its commencement exercises April 29.

This college has inaugurated an advance step in the State of Illinois in the matter of final examinations.

By a vote of its Faculty and also of the graduating class, the Illinois State Board of Dental Examiners was requested to conduct the final examinations of the graduating classes. The examinations of the State Board were an oral in addition to the regular written examinations conducted by the Faculty.

---

Dr. W. C. Wardlaw says that dentists are too often remiss in ability to satisfactorily answer the unwelcome questions of intelligent patients as to "why" the teeth decay, but whip the devil around the stump with indefinite generalities, or cover up an inglorious retreat with a flow of technical terms.

Where a cavity extends both up the neck of the tooth and on the labial surface of the crown, Dr. A. H. Thompson selects a piece of gum tooth having the proper color for both gum and crown portion, and inserts an inlay, restoring the whole to a perfectly natural appearance. If the color of both gum and tooth cannot be matched from a single tooth, each can be matched separately and dovetailed together, the joint being imperceptible if accurately ground.

---

Dr. T. E. Weeks says that at thirteen, if injury is inflicted at the distal ends of the dentinal fibrils, the nerve-endings, it is transmitted along the fibrils to the sensorium. The water in the tubuli, which is a constituent of the fibril, and which also surrounds it, is essential to this transmission of sensation. In proportion as the water is removed sensation is modified. Hence the adoption of different methods of dehydration for the control of sensitiveness of dentine, either by raising the temperature by means of hot air etc., or lowering it through cold applications. The rapid motion of a keen instrument on sensitive dentine is painless, but the instrument should be one that will free itself of chips, and be small, thus having less friction.

---

Dr. Peterson, Iowa, has, with the greatest satisfaction to himself, replaced unsightly, glaring gold fillings, in the neck of the anterior teeth of his wife, with porcelain inlays made from good English teeth, in which he was fortunate in finding exactly the right shade. He had inserted the gold fillings as the only thing to be done in the case, but deplored them as "a vulgar advertisement of his own work." He thought it much easier to grind a piece of ready-made tooth than to fire up a furnace and bake a small filling.

---

The Fourteenth Annual Meeting of the New Hampshire Dental Society will be held in Concord, commencing Monday evening, June 16, and continuing till Wednesday noon of the 18th. An interesting programme will be issued to members of the profession in this State in due time, and all are cordially invited to attend.

The Board of Censors will meet on Monday evening, June 16, in connection with the Society.

Edward B. Davis, *Secretary.*

